# Reorganization of functional and directed corticomuscular connectivity during precision grip from childhood to adulthood

**DOI:** 10.1038/s41598-021-01903-1

**Published:** 2021-11-24

**Authors:** Mikkel Malling Beck, Meaghan Elizabeth Spedden, Jesper Lundbye-Jensen

**Affiliations:** Department of Nutrition, Exercise and Sports, University of Copenhagen, Nørre Alle 51, 2200 Copenhagen N, Denmark

**Keywords:** Neuroscience, Physiology

## Abstract

How does the neural control of fine movements develop from childhood to adulthood? Here, we investigated developmental differences in functional corticomuscular connectivity using coherence analyses in 111 individuals from four different age groups covering the age range 8–30 y. EEG and EMG were recorded while participants performed a uni-manual force-tracing task requiring fine control of force in a precision grip with both the dominant and non-dominant hand. Using beamforming methods, we located and reconstructed source activity from EEG data displaying peak coherence with the EMG activity of an intrinsic hand muscle during the task. Coherent cortical sources were found anterior and posterior to the central sulcus in the contralateral hemisphere. Undirected and directed corticomuscular coherence was quantified and compared between age groups. Our results revealed that coherence was greater in adults (20–30 yo) than in children (8–10 yo) and that this difference was driven by greater magnitudes of descending (cortex-to-muscle), rather than ascending (muscle-to-cortex), coherence. We speculate that the age-related differences reflect maturation of corticomuscular networks leading to increased functional connectivity with age. We interpret the greater magnitude of descending oscillatory coupling as reflecting a greater degree of feedforward control in adults compared to children. The findings provide a detailed characterization of differences in functional sensorimotor connectivity for individuals at different stages of typical ontogenetic development that may be related to the maturational refinement of dexterous motor control.

## Introduction

Humans develop their dexterous abilities from childhood though adolescence to adulthood^1–3^. Efficient functioning of sensorimotor neural networks is a prerequisite for fine control of the hands and fingers during motor tasks. Development of corticomuscular control mechanisms could potentially contribute to improvement of skilled capacity as we age^4^, but little is known about the actual neurophysiological mechanisms that lead to improved dexterity from childhood to adulthood.

The human central nervous system (CNS), including the descending and ascending pathways between the brain and spinal cord, is continuously shaped during ontogenetic development. This is e.g. reflected in gradual increases in white matter integrity in the corticospinal tract^5,6^ and an increase in the central conduction velocity of both ascending and descending pathways measured via brain and muscle responses to synchronous activation of a peripheral nerve by electrical stimulation and the corticospinal system using transcranial magnetic stimulation (TMS) of the primary motor cortex (M1), respectively^7–9^. These maturational adaptations can shape the passing and processing of information in functional neural networks^10^ and thereby likely also affect patterns of connectivity between brain and spinal cord. However, little is actually known about developmental differences in task-related functional connectivity in corticomuscular networks. Oscillatory coupling between neuronal populations within the CNS may represent a processing strategy allowing efficient neural interactions^11^. During voluntary motor tasks, oscillatory activity in parts of the cerebral cortex dedicated to sensorimotor functions correlates with similar rhythms in the contralateral contracting muscles in adult humans^12^ and non-human primates^13^. These patterns of functional connectivity can be captured by measures of corticomuscular coherence reflecting frequency-domain linear correlations between brain activity obtained by magneto- or electroencephalography (M/EEG) and muscle activity from electromyographic (EMG) recordings. Coherence between the rhythmic signals of the brain and muscle is particularly prominent in the beta band (15–30 Hz) during steady voluntary muscle contractions^12,14^.

Patterns of correlated oscillatory activity in the corticomuscular system have been found to emerge during late childhood and to steadily increase in strength until early adulthood^15–19^. From a functional perspective, corticomuscular coherence during steady-state muscle contractions has been associated with greater control over motor output in adults, as evidenced by smaller fluctuations in the force produced^20^ and less variable muscle activity^21^. These studies demonstrate a gradual strengthening of oscillatory corticomuscular interactions during development that could relate to the ability to control fine motor output.

Developmental differences in the functional coupling between brain and muscle activity have been studied for several different muscles including the hand^18^, forearm^15^ and ankle^16^ muscles on either right or left side of the body. Levels of coherence have been shown to follow a proximal–distal gradient, with higher magnitudes observed for the latter in adults^22^, but it is currently unknown whether there are differences in coherence between the dominant and non-dominant limb and whether this may change during development.

Early studies advocated that corticomuscular coherence is mediated by beta-range oscillations descending from cortex via the corticospinal tract to the spinal cord to drive the activity of motor neurons^23,24^, but emerging evidence suggests that this interpretation may be somewhat simplified^25^. For example, administration of pharmacological agents can alter the power of cortical beta-range oscillations or corticomuscular coherence selectively; a result that is at odds with the notion of an exclusive descending and efferent origin of coherence (i.e. a pure feedforward system)^26,27^. Instead, sensory (ascending) inputs to sensorimotor cortices might also affect levels of corticomuscular coherence^24,28,29^, thus forming a sensorimotor loop between the cortex and periphery that may be relevant for binding of motor commands and their sensory consequences. Coherence captures the statistical association between signal pairs in the frequency domain, quantifying the degree of functional connectivity, but not the direction. In contrast, measures of *directed* coherence allow a dissection of descending (from cortex to muscle) and ascending (from muscle to cortex) contributions to coherence based on time lags between the two signals of interest^30^. From a developmental perspective, such information is highly relevant, as it may reveal fundamental insights into potential shifts in the neural control mechanisms that are used to guide behavior. To date, potential developmental differences in directed corticomuscular coherence have been largely unexplored. In a recent study, we demonstrated that the degree of descending coherence (from cortex to muscle) increased at the expense of ascending coherence (from muscle to cortex) as a function of age from childhood through adolescence. These increased levels of descending coherence could reflect increased reliance and/or efficiency of feedforward control guiding motor behavior with a concomitant reduction in the importance of ascending feedback^16^. This was the first study to characterize developmental differences in directed corticomuscular coherence, but more studies are needed to confirm this. Furthermore, EEG was recorded from a single electrode (over Cz) and focus was on the control of the ankle muscles. Therefore, age-related differences in directed connectivity between brain and contracting finger muscles—potentially reflecting a functional reorganization of coherent corticomuscular networks from childhood to adulthood—have yet to be fully explored.

Here, we investigated functional corticomuscular connectivity during a uni-manual force-tracing task involving steady and precise control of the force generated by the intrinsic hand muscles with the aim of characterizing age-related differences from childhood to adulthood and explore effects of hand dominancy in a large sample of typically developed individuals. Based on recent results^16^, we hypothesized that the amount of corticomuscular coherence would increase with age for both the dominant and non-dominant hand and that the level of descending corticomuscular coherence (cortex-to-muscle) would be greater for older compared to younger individuals.

## Materials and methods

### Participants

One hundred fourteen individuals were recruited to participate in the experiment. Participants received thorough oral and written information and informed consent was obtained from participants (> 18 y) or their parents (< 18 y) prior to enrollment. The study was approved by the regional ethical committee of the Greater Copenhagen Area (‘De Videnskabsetiske Komiteer for Region Hovedstaden’; protocol number: H-17019671) and adhered to the principles set out in the declaration of Helsinki II. EEG and motor performance (i.e. motor precision) data from the dominant hand in a subset of the sample has already been included in a previous paper, in which we assessed age-related differences in cortico-cortical connectivity (using Dynamic Causal Modelling) and motor performance^31^. Here, we present data on corticomuscular coherence and task performance for the same task from both the dominant and non-dominant hand. Participants’ handedness were determined by the Edinburgh handedness inventory^32^ and levels of physical development were characterized using a sketched version of the Tanner scale^33,34^.

### Experimental procedure

**Figure 1:**
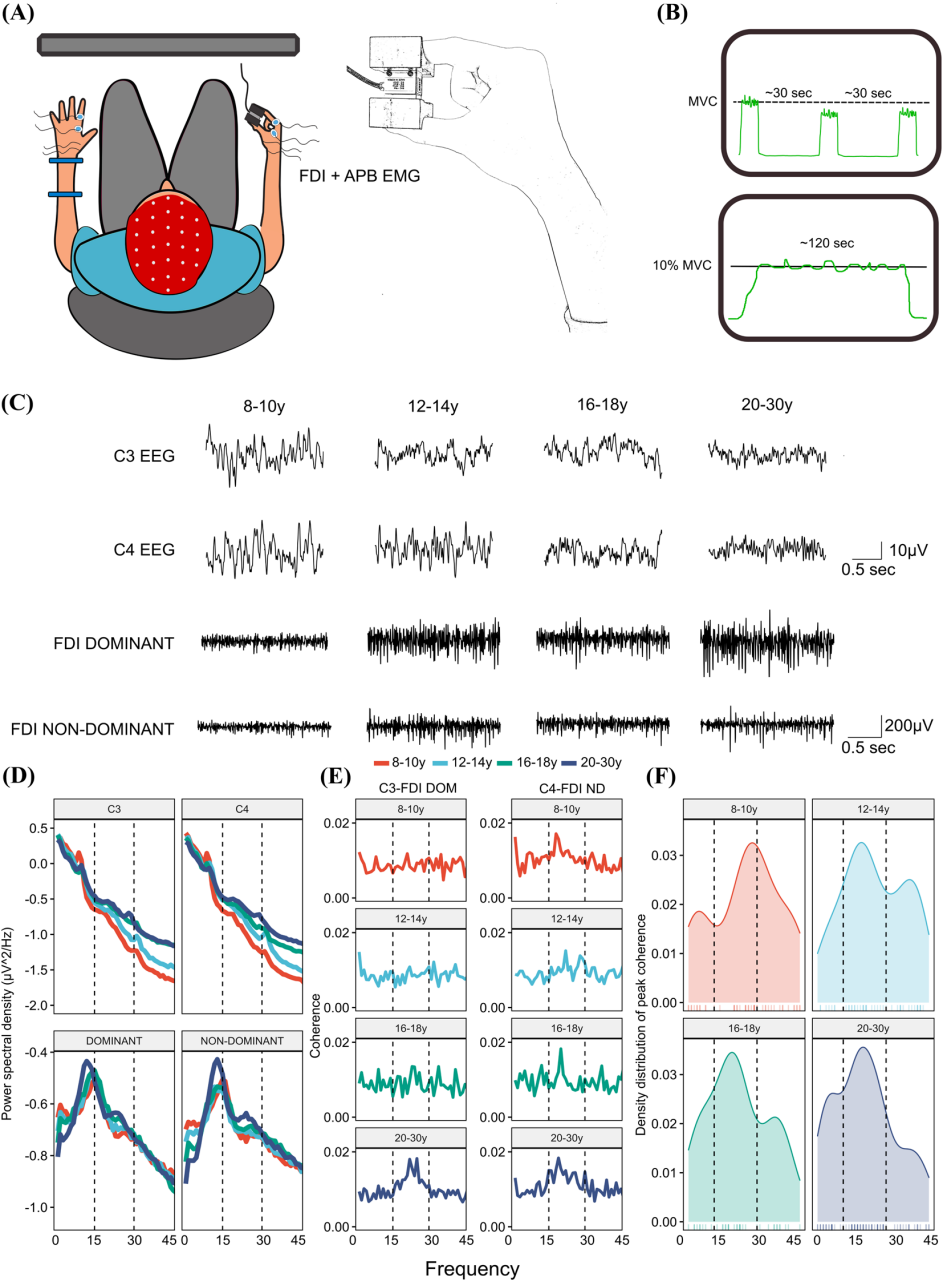
Experimental setup and scalp EEG and EMG data. EEG was recorded from 64-channels and EMG was obtained from the FDI and APB muscles of both the dominant and non-dominant hand. Participants initially performed three maximal voluntary contractions (MVC) on the non-dominant hand, before performing the force-tracing task in which participants were asked to maintain a steady force between the index and thumb corresponding to 10% of MVC. Visual feedback was provided as a real-time trace of the applied force (**A** and **B**). In (**C**) sensor EEG data and FDI EMG are presented from representative individuals from electrode C3 and C4 (located approximately above primary motor cortex) and the dominant and non-dominant hands. (**D**) Presents group-averaged sensor-level power spectral densities from the C3 and C4 EEG electrodes alongside the dominant and non-dominant FDI EMG. (**E**) Displays average coherence spectra from each of the four age groups. (**F**) Displays group-level density plots of the distribution of peak coherence frequency in the 5–45 Hz frequency range. The dashed vertical black lines in (**D**–**F**) mark the beta-band (15–30 Hz). Please note the common legend used for the plots in DEF. DOM = dominant hand; ND = non-dominant hand; FDI = First dorsal interosseous; APB = Abductor pollicis brevis.

EEG and EMG recordings were acquired while participants performed a tonic force-tracing task with both the dominant and the non-dominant hand. The procedure for each hand was similar. Participants sat in a chair in front of a 27″ computer monitor. Participants initially performed three maximal voluntary contractions (MVC). Then, a horizontal target line corresponding to 10% MVC was presented in the middle of the screen in front of them. Participants were asked to apply force to a load cell (Dacell, AM210, Dacell Co. Ltd., Korea) located between their index finger and thumb in a precision grip and match the force produced to a horizontal target line (Fig. 1A,B). The force produced was amplified 100× and low-pass filtered at 10 Hz (Dacell, AM210, Dacell Co. Ltd., Korea) before being digitized at 1000 Hz (CED1401, Cambridge Electronic Design Ltd, Cambridge, UK) and fed back to the participant as a real-time trace of the applied force (Fig. 1B). The task was performed for ~ 120 s on each hand starting with the non-dominant hand. This specific task was chosen because periods of steady contraction in the precision grip are characterized by corticomuscular coherence in the beta-range (15–30 Hz) in adults. Performance in the force-tracing task was quantified as motor precision and motor variability. Motor precision was defined as the root mean squared error (RMSE) from the horizontal target line (i.e. deviations from ‘optimal’ performance. This score was reverse coded (multiplied by − 1) to reflect motor precision (i.e. inverse error). Variability of motor output was indexed by computing the coefficient of variation (CV) of the force produced. In a subsequent control experiment, we recruited 15 adults who were naïve to the task. These individuals completed the same experiment as described above, but with the order of tasks reversed (i.e. the task was performed with the dominant hand first). This was done to assess potential order effects.

### Data acquisition

EEG and EMG were sampled using a BioSemi amplifier system (BioSemi, Amsterdam, The Netherlands) using ActiView software (v7.07) installed on a PC. EEG was obtained from 64 sensors placed in a standard electrode cap with the 10/20 layout (BioSemi, Amsterdam, The Netherlands), and EMG data was obtained from 4 pairs of electrodes positioned over the first dorsal interosseous (FDI) and abductor pollicis brevis (APB) muscle on both the dominant and non-dominant hand in a belly-tendon montage (Fig. 1A; left). Data was sampled as raw signals at 2048 Hz while participants performed the force-tracing task described above. Before performing the task, participants were instructed to keep their neck and face relaxed to avoid excessive muscle activity in the EEG. Furthermore, we instructed participants on keeping the hand not performing the task still and resting on the table. Electrode offsets were < 30 mV for all EEG sensors prior to initiating the recordings. The common mode sensor (CMS) and the driven right leg (DRL) electrodes provided the reference during the data acquisition.

### EEG and EMG preprocessing

EEG and EMG data was preprocessed in EEGLAB (v14.1.1b)^35^ in Matlab R2017b. All subsequent steps were performed on files from both the dominant and the non-dominant hand. Raw data files were imported using the Biosig toolbox. Data was visually inspected and time periods in the EEG exhibiting high amplitude fluctuations due to e.g. muscle activity were removed. EEG data was separated from EMG data. Then, EEG data was band-pass filtered from 0.5 to 48 Hz (automatic filter-order as per EEGLAB defaults) and subsequently downsampled to 256 Hz. EEG data was visually inspected and bad channels displaying excessive noise were removed prior to re-referencing sensor activity to average reference (average of 1.99 ± 1.65 channels removed). Finally, independent component analysis (ICA) was performed (‘*runica*’ algorithm) to decompose the EEG data into independent components. Components reflecting eye-blinks and/or horizontal eye movements were removed from the data by visual inspection before the removed channels were interpolated using the default spherical interpolation procedure. To limit the total number of analyses, we chose to focus on the EMG data from the FDI and not the APB muscle. This was filtered between 5 and 120 Hz and downsampled to 256 Hz. Next, data files were converted to Statistical Parametric Mapping (SPM) formatted files and epoched in non-overlapping segments with an epoch-length of 1-s using SPM12 (v. 7490). Next, default electrode positions were registered. Electrode positions were flipped across the midline in the sagittal plane for left-hand dominant individuals (n = 8) to enable group-level comparisons.

### Data analysis

Subsequent steps were performed on files from both the dominant and the non-dominant hand. The first descriptive analyses were performed at the sensor level. As a first step, we computed power spectral densities from the C3 and C4 electrode and the FDI EMG from both the dominant and non-dominant hand (please see Fig. 1C for raw data time series). Power spectra were computed using a Finite Fourier Transformation (FFT) to represent the signal in the frequency domain (Fig. 1D). The choice of electrodes was motivated by the fact that corticomuscular coherence generally is topographically located contralateral to the contracting muscles at electrodes overlying primary sensorimotor regions^36^.

Next, we aimed to characterize the frequency distribution of corticomuscular coherence. This was done by two complementary approaches as described below. First, we computed coherence spectra for each individual. Coherence between two signals (x,y) at a given frequency *ω* C_xy_(*ω*) is represented as the squared magnitude of the cross-spectrum between the two signals (f_xy_) divided by the product of the two auto spectra (f_xx_(*ω*) and f_yy_(*ω*)), with values bound between 0 and 1:

$${\left|{C}_{xy}\left(\omega \right)\right|}^{2}=\frac{{\left|{f}_{xy}(\omega )\right|}^{2}}{{f}_{xx}\left(\omega \right)*{f}_{yy}\left(\omega \right)}$$

Coherence values of zero at a given frequency indicate a lack of statistical association between the two signals, whereas values of one represent an ideal linear correlation in the frequency-domain. Time series from the C3 and C4 electrodes and the EMG from the FDI muscle were extracted and subjected to this analysis. EMG signals were full-wave rectified^37^ and both EEG and EMG data was linearly de-trended and normalized to unit variance. Auto and cross-spectra used for the coherence analysis were estimated by FFTs of non-overlapping segments of 1-s (256 samples, frequency resolution of 1 Hz) and power and coherence spectra were visually inspected to assess data quality. Next, individual coherence spectra were averaged on the group-level to provide a descriptive summary of group data (see Fig. 1E for group-averaged coherence figures on the sensor level). These analyses were performed using the Neurospec toolbox (v 2.11)^38^. Second, we also extracted the frequency value where coherence was maximal from the individual coherence spectra and plotted the distribution of peak frequencies for each age group. In accordance with earlier studies, these descriptive analyses revealed that, for all age groups, peak coherence was most commonly observed within the beta band (15–30 Hz) (Fig. 1E,F).

After having established the frequency distribution of corticomuscular coherence at the sensor-level, we next turned to localize brain sources that were coherent with muscle activity in the active FDI muscle. We focused our analysis on the beta band because (1) our sensor-level data suggested that this was the most common frequency band in which peak coherence occurred across the population studied; and (2) previous studies have shown that corticomuscular coherence is particularly pronounced in the beta-range during steady-state motor output (as reviewed in Mima and Hallett^36^; van Wijk et al.^39^; Grosse et al.^40^). Cortical sources that were coherent with muscle activity in the active FDI muscle were identified using Dynamic Imaging of Coherent Sources (DICS) beamforming^41^ implemented in the Data Analysis in Source Space (DAiSS) toolbox in SPM12. DICS uses the cross-spectral density (CSD) matrix combined with a forward model to localize coherent sources of activity in the brain with a peripheral signal (here, the FDI-EMG channel). Although DICS was originally designed for MEG data, it has been found to yield physiological sensible results for EEG data as well^42^. Beamforming techniques use an adaptive spatial filter at each point of a search grid of the brain to maximize the point source activity and attenuate the activity originating from other sources. These filters are a linear transformation of the lead fields and the CSDs. Lead fields were computed using the Boundary Element Model (BEM) based on the template MRI provided in SPM12^43^. The template MRI was used as individual structural MRIs were not obtained. The source grid resolution was set to 5 mm. Results were stored as individual volumetric images. Individual images were smoothed using a Gaussian kernel (10 × 10 × 10 mm) and individually thresholded to include voxels that were two times larger than the standard deviation above the mean coherence across all voxels. Grand averages were computed for the dominant and non-dominant hand from the smoothed and individually thresholded images from all participants. Next, using Linear Constrained Minimum Variance (LCMV) beamforming^44^ embedded in DAiSS in SPM12, individual time-series of source activity were reconstructed as a ‘virtual electrode’ from the grand-average cortical location displaying beta-range peak coherence (MNI coordinates for the dominant hand: − 28; y = − 6; z = 58; and the non-dominant hand: x = 30; y = − 2; z = 48)^45^. This was done to facilitate comparisons between different groups. Subsequently, coherence was estimated from the source reconstructed cortical time-series and the EMG data from the active FDI muscle using a similar approach to the one described above on 120.8 ± 4.4 s of data from each participant. This analysis was performed to quantify the (1) proportions of individuals displaying significant coherence (2) the peak and the area of beta-band coherence and (3) the directionality, i.e. magnitudes of descending (Cortex-to-EMG) and ascending (EMG-to-cortex) components of coherence in the beta band. Significance of corticomuscular coherence for each individual was determined based on a NULL hypothesis of uncorrelated signal pairs (Gaussian noise) by computing the upper 95% confidence limit as per^38^:

$$1-{\alpha }^{\frac{1}{N-1}}$$

where α is the confidence limit and N is the number of non-overlapping data segments in each recording. As we were interested in determining whether individuals displayed significant coherence at any frequency within the beta-range, we corrected the α-level to reflect the 15 comparisons (0.05/15 = 0.0033). Directed coherence was identified by filtering the data with a pre-whitening filter prior to estimating coherence^30^. In doing so, each of the auto-spectra approaches white noise, but the correlation between the two signals is preserved. As a result, the product of the denominator of the coherence function equals 1, and the coherence can be expressed as the magnitude squared of the pre-whitened cross-spectrum. By using an inverse Fourier transform of the pre-whitened cross-spectrum, one can obtain the time-domain correlation function. This enables a non-parametric decomposition of forward (descending), backwards (ascending) and zero-lagged contributions to coherence at each frequency based on the time lag between the two signals (for a detailed description of the method please see^30^). We restricted this analysis to the beta-range and computed band-limited scalar measures of associations between signals in the descending and ascending directions as well as instantaneous (zero-lagged) association. As zero-lagged contribution to coherence may reflect common (non-physiological) activity present in both EMG and the source reconstructed data^46^ this was discarded.

### Statistical analyses

All statistical analyses were performed in R studio (v. 4.0.0)^47^. Potential differences in measures of performance or coherence due to age group and hand used were investigated using linear mixed effect models. Linear mixed effects models were chosen as they allow fitting models on incomplete datasets (see “Results” section). Individual models with the dependent variables were fitted with age group and hand as the independent fixed variable with an interaction (Group × Hand). Order of tasks was added as an additional covariate to account for the fact that some individuals were recruited for a control experiment in which the order of the task was reversed (dominant before non-dominant hand). To account for inter-individual differences in coherence and the repeated measures design we added ‘participants’ as random intercepts. For the analysis of directed coherence (magnitude of descending and ascending coherence components), we also added total beta-band coherence area or coherence peak as additional covariates to account for the fact that directed coherence components scale with total levels of beta-range area and peak coherence (which co-vary with age group) (see supplementary Fig. S1). Models were fitted using the *lme4* package^48^ and p-values for main and interaction effects were obtained using the *lmerTest* package^49^ using the Satterthwaite’s method for estimating degrees of freedom. To evaluate the size of the reported effects, we additionally calculated Cohen’s d using the group or hand averages and pooled standard deviations for the statistically significant comparisons. An effect size of 0.2–0.5 was considered small; 0.5–0.8 moderate and effect sizes of ≥ 0.8 were considered as large.

Associations between measures of coherence and performance were also investigated using linear mixed models. For these analyses, age group was added as a covariate and ‘participants’ added as random intercepts. For all analyses using linear mixed effect models, we checked for normality of residuals visually through qqplots, and log transformations were applied to the dependent variable if this was not fulfilled (this was deemed to be the case for the variables motor precision; motor variability; the area of beta coherence; the peak of beta coherence). This data is also presented as log-transformed values in the graphs. For multiple comparisons, we controlled for the false discovery rate (FDR) and computed 95% confidence intervals using the *emmeans* package. This comprised the group-wise comparisons of (1) motor performance; (2) area and peak coherence; (3) descending and ascending magnitudes of coherence.

## Results

Participants generally completed the task as intended with both the dominant and non-dominant hand. Some data was excluded due to excessive movements artefacts, an inability to understand and perform the task as intended or technical malfunctions (a total of 30/250 raw data files were excluded; 16 from the dominant hand and 14 from the non-dominant hand). This resulted in a total of 109 EEG-EMG data files that were analyzed from the dominant hand and 111 data files that were used for the analysis for the non-dominant hand. Please also note that data files from the force-tracing task were not saved following acquisition for seven participants due to technical issues. Table 1 provides a summary of participant characteristics.

**Table 1:** Participant characteristics.

	8–10 y (n = 24)	12–14 y (n = 23)	16–18 y (n = 20)	20–30 y (n = 45)
Age (years)	9.05 ± 0.61	13.05 ± 0.53	17.20 ± 0.57	24.84 ± 2.67
Sex (male/female)	14/9	12/11	10/10	20/25
Tanner developmental stage	1.12 ± 0.27	2.56 ± 1.01	4.63 ± 0.54	5 ± 0
Handedness (right/left dominant)	18/6	21/2	19/1	45/0

Continuous values are presented as mean values ± standard deviation of the mean (SD). Categorical values are presented as counts.

### Differences in performance in precision grip task due to age group and hand used

**Figure 2:**
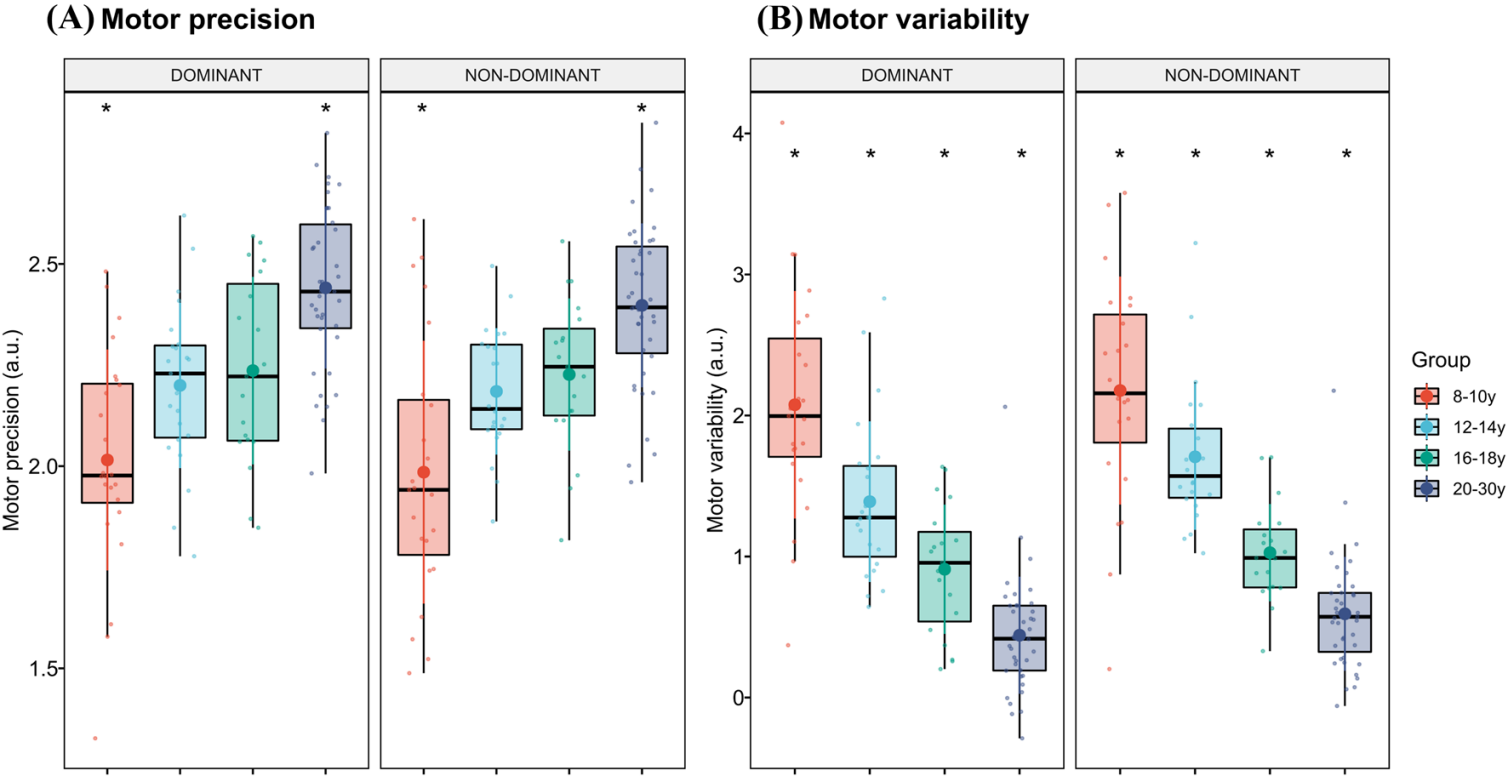
Motor performance. Box plots with individual data points displaying (**A**) precision performance and (**B**) motor variability in the force-tracing task for the different age groups divided by hand used (N = 104 for dominant hand; N = 103 for non-dominant hand). Big filled circle reflects mean value and adjoining lines colored by group reflect standard deviations (SD). *Signifies a significant difference from the remaining groups across hands (p < 0.05).

Participants were instructed to match their force output in a precision grip to a visually presented horizontal target-line for 2 min. Performance in the tonic precision grip task for the different age groups for both the dominant and non-dominant hand is presented in Fig. 2. Please note that precision performance for the dominant hand has already been presented elsewhere for a subset of the participants^50^. For motor precision, a main effect of age group was found (F = 20.2; P < 0.001). Subsequent contrasts revealed that individuals in the 20–30 y age group significantly outperformed the other groups (β_20–30y vs 8–10y_ = 0.43 ± 0.06; 95% CI [0.28 0.58]; P < 0.001; Cohen’s d = 1.65; β_20–30y vs 12–14y_ = 0.22 ± 0.06; 95% CI [0.09 0.39]; P < 0.001; Cohen’s d = 1.15; β_20–30y vs 16–18y_ = 0.19 ± 0.06; 95% CI [0.03 0.35]; P = 0.002; Cohen’s d = 0.88). Additionally, individuals aged 8–10 also performed significantly worse compared to the 12–14 y (β_8–10y vs 12–14y_ = − 0.19 ± 0.06; 95% CI [− 0.35 − 0.03]; P = 0.002; Cohen’s d = 0.79) and the 16–18 y (β_8–10y vs 16–18y_ = − 0.24 ± 0.06; 95% CI [− 0.41 − 0.07]; P < 0.001; Cohen’s d = 0.91). No significant effect of task order (F = 0.15; P = 0.69), hand (F = 1.24; P = 0.27) or interactions between hand used and age group were found (F = 0.31; P = 0.82).

For motor variability, a main effect of age group was found (F = 53.0; P < 0.001). Subsequent contrasts revealed a linear decrease in motor variability across age groups. Variability was lower in the 20–30 y age group compared to all other groups (β_20–30y vs 8–10y_ = − 1.66 ± 0.14; 95% CI [− 2.04 − 1.28]; P < 0.001; Cohen’s d = 1.29; β_20–30y vs 12-14y_ = − 1.10 ± 0.14; 95% CI [− 1.48 − 0.72]; P < 0.001; Cohen’s d = 1.17; β_20–30y vs 16–18y_ = − 0.48 ± 0.15; 95% CI [− 0.88 − 0.08]; P = 0.002; Cohen’s d = 0.83); lower in the 16–18 y age group compared to the 12–14 y (β_16–18y vs 12–14y_ = − 0.62 ± 0.16; 95% CI [− 1.04 − 0.22]; P < 0.001; Cohen’s d = 0.87) and the 8–10 y groups (β_16–18y vs 8–10y_ = − 1.18 ± 0.15; 95% CI [− 1.60 − 0.77]; P < 0.001; Cohen’s d = 1.15); and, finally, lower for the 12–14 y age group compared to the 8–10 y age group β_12–14 vs 8–10y_ = 0.56 ± 0.15; 95% CI [− 0.95 − 0.16]; P < 0.001; Cohen’s d = 0.72). A significant main effect was also found for the effects of hand (F = 12.5; P < 0.001), driven by a lower degree of motor variability on the dominant hand compared to the non-dominant hand (β_dominant vs non-dominant hand_ = − 0.16 ± 0.04; 95% CI [− 0.25 − 0.07]; P < 0.001; Cohen’s d = 0.20). No significant effect of task order (F = 0.81; P = 0.37) or interactions between hand used and age group was found (F = 0.94; P = 0.42).

### Differences in corticomuscular coherence due to age group and hand use

To determine frequency patterns of corticomuscular coherence across age groups we averaged individual coherence spectra (Fig. 1E) and extracted individual peak frequencies of coherence (Fig. 1F). In accordance with earlier studies, these descriptive analyses revealed a predominance of beta-range corticomuscular coherence across the entire sample. This led us to restrict our source reconstruction analysis to the beta-band.

**Figure 3:**
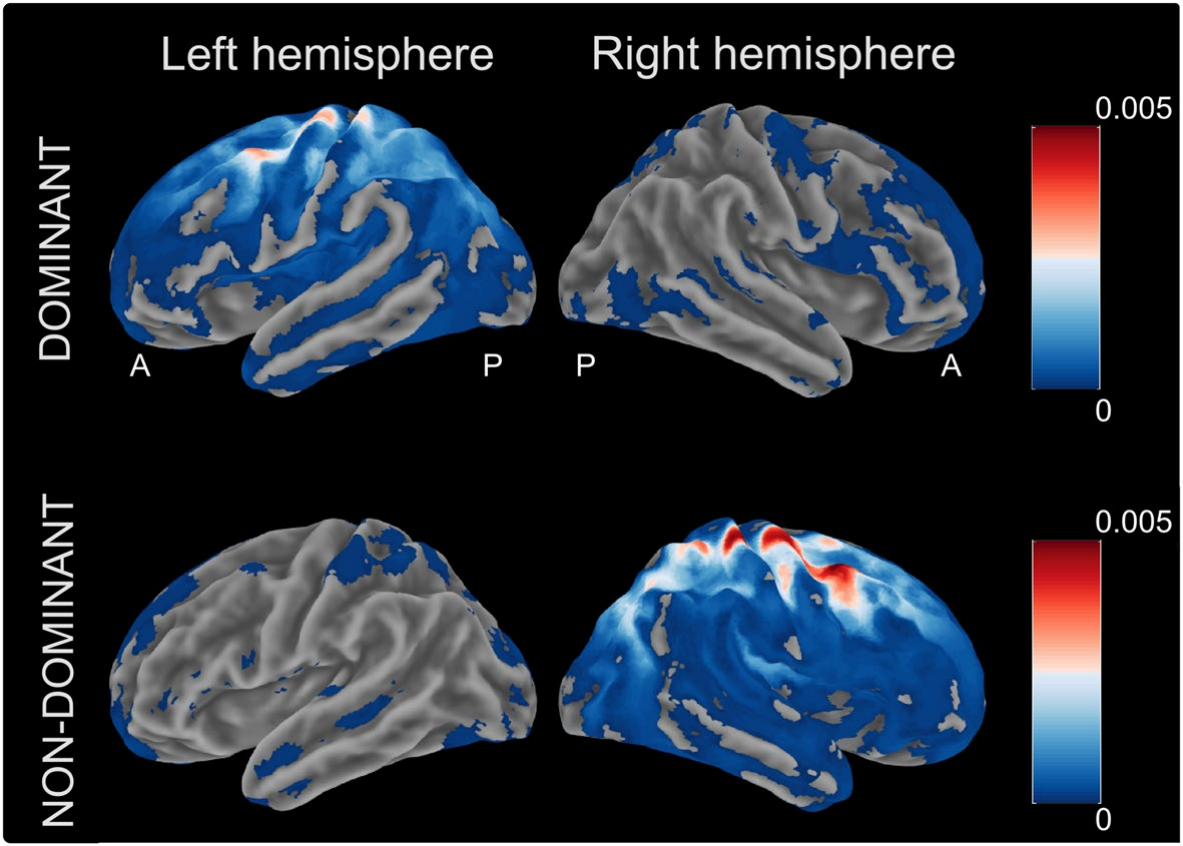
Dynamic imaging of coherent sources. Displays age-related differences in localization of beta-range peak coherence with active FDI muscle for the dominant and non-dominant hand. Sources are displayed on the template MNI brain provided in the visualization tool MRIcron at z = 60. A = Anterior; P = Posterior; L = Left; R = Right.

To localize brain sources displaying coherent activity with the FDI-EMG channel a DICS analysis was performed. The group-level results are shown as volumetric images in Fig. 3. Generally, cortical sources displaying maximal coherence with FDI-EMG activity were located in the contralateral hemisphere just anterior or posterior to the central sulcus for both the dominant and non-dominant hand.

Reconstructed time-series of source activity were extracted for each individual, and coherence analysis was performed. We quantified the numbers of individuals displaying beta-range coherence peaks exceeding the upper confidence limit (Table 2). Across the entire sample, 19.5% of individuals displayed significant coherence in the beta-band, and the proportion seemed to scale with age.

**Table 2:** Percentage of individuals displaying peak corticomuscular coherence values exceeding the corrected upper confidence limit (α = 0.0033) in the beta-band (15–30 Hz) by group and hand.

	Dominant hand (%)	Non-dominant hand (%)
8–10 y	8.3	4.2
12–14 y	13.0	13.6
16–18 y	10.5	35
20–30 y	20.9	31.1

**Figure 4:**
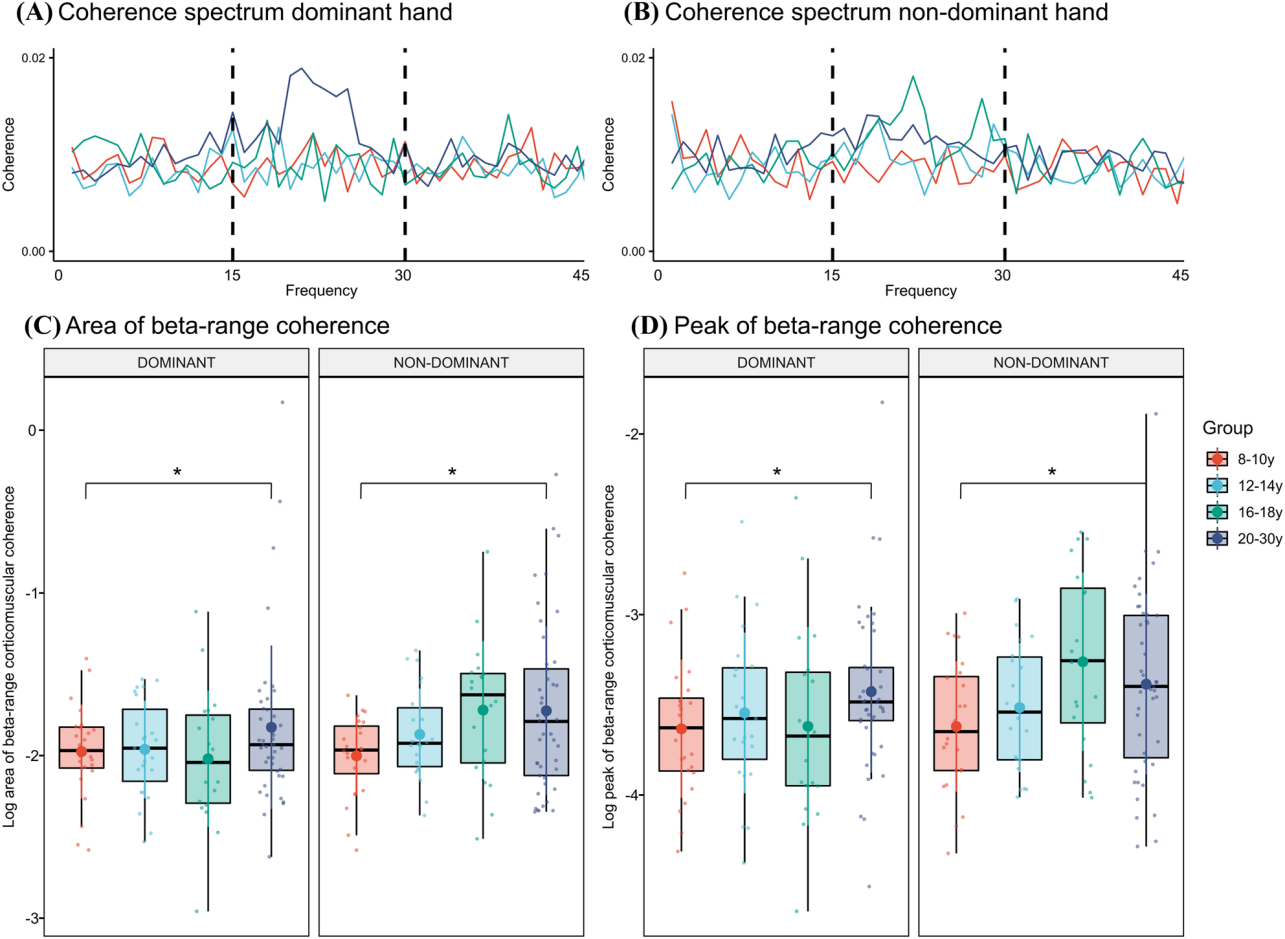
Beta-band corticomuscular coherence. Average coherence spectra for the dominant (**A**) and non-dominant hand (**B**) alongside box plots with individual data points and means displaying age-related differences in the log area (**C**) and peak (**D**) of beta-band corticomusclar coherence between cortical source activity and EMG activity from FDI muscles on the dominant and non-dominant hand. Statistically significant differences from the 8–10 y group are marked by * (p < 0.05).

We compared whether significant differences were present for beta-range coherence area as a function of age group and hand (Fig. 4A–C). The analysis revealed an effect of age group (F = 3.27; P = 0.024). As can be seen in Fig. 4A–C, this effect was driven by greater levels of beta-range coherence in the 20–30 y group compared to the 8–10 y group (β_20–30y vs 8–10y_ = 0.27 ± 0.09; 95% CI [0.03 0.51]; P = 0.020; Cohen’s d = 0.53). There were no significant differences between the remaining groups (range of p-values: 0.11–0.67). We also found a significant effect of hand (F = 5.13; P = 0.025) which reflected that the area of beta-range coherence was greater for the non-dominant hand compared to the dominant hand (0.12 ± 0.05; 95% CI [0.01 0.21]; P = 0.026; Cohen’s d = 0.26). No significant effect of order (F = 2.40; P = 0.12) or interactions between age group and hand were seen (F = 1.57; P = 0.20).

Comparable results were found for beta-range peak coherence. A significant effect of age group was found (F = 2.92; P = 0.037), and this was driven by greater coherence peak values in 20–30 y than in 8–10 y (β_20–30y vs 8–10y_ = 0.27 ± 0.09; 95% CI [0.01 0.51]; P = 0.033; Cohen’s d = 0.49) (Fig. 4D). No significant differences were found between the remaining groups (range of p-values: 0.17–0.46). No significant effects of hand (F = 2.92; P = 0.076), order (F = 1.26; P = 0.27) or interactions between age group and hand were found (F = 1.60; P = 0.19). Notably, similar age-related and hand effects were seen when comparing coherence peak values at the individual peak location of coherence (see supplementary results, Fig. S2).

**Figure 5:**
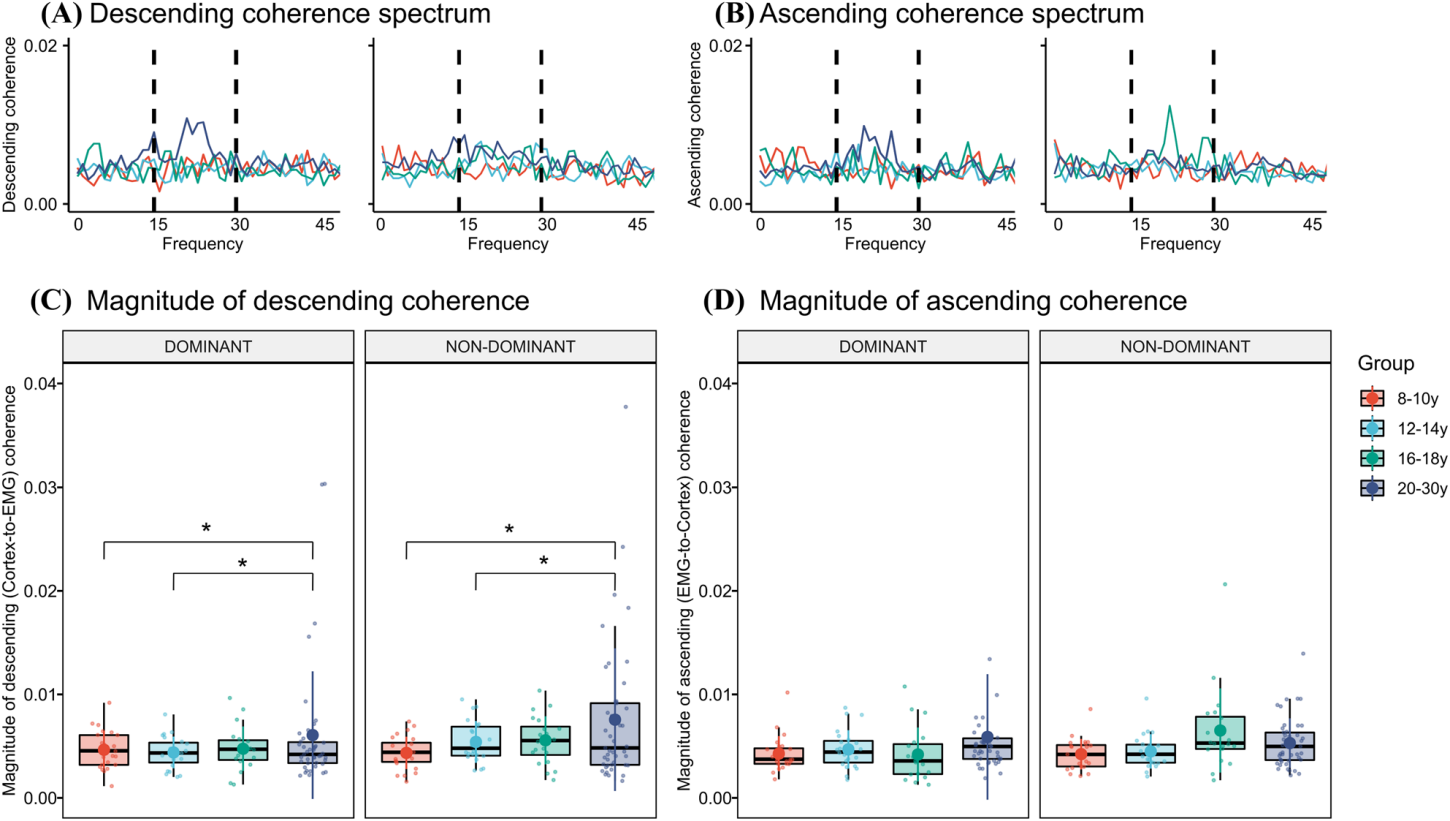
Directed corticomuscular coherence. Group-averaged coherence spectra for the descending (**A**) and ascending (**B**) components of coherence on the dominant (left spectrum) and non-dominant (right spectrum) hand. Box plots with individual data points and mean level displaying age-related differences in the descending (**C**) and ascending (**D**) amplitudes of beta-band corticomusclar coherence between cortical source and active FDI muscles on the dominant and non-dominant hand. Statistical significant differences from the 8–10 y group is marked by * (p < 0.05).

For directionality components, we found a significant effect of age group on the levels of descending (Cortex-to-EMG) coherence (F = 3.60; P = 0.016). Specifically, individuals in the 20–30 y group had significantly larger magnitudes of descending coherence compared to the 8–10 y group (β_20–30y vs 8–10y_ = 0.003 ± 0.001; 95% CI [0.0003 0.006]; P = 0.022; Cohen’s d = 0.49) and the 12–14 y group (β_20–30y vs 12–14y_ = 0.003 ± 0.001; 95% CI [0.0002 0.005]; P = 0.044; Cohen’s d = 0.40), but not between any of the other groups (range of p-values: 0.06–0.86) (Fig. 5A,C). The main effect of age group persisted when controlling for peak beta-range coherence (F = 3.23 = 0.025), but it became non-significant when controlling for beta-range coherence area (F = 1.56, P = 0.20). No main effect of hand was found (F = 2.50; P = 0.12), and no interaction between age group and hand was observed (F = 0.87; P = 0.46). For the ascending component (EMG-to-cortex) (Fig. 5B,D), we found no significant effects of age group (F = 2.04; P = 0.11), hand (F = 0.62; P = 0.43) or interactions between age group and hand (F = 1.82; P = 0.15).

As a final step, we used regression models to determine potential statistical dependencies between measures of beta-range coherence and motor performance (quantified as motor precision and motor variability). We did not find any significant associations between the included measures of coherence and motor precision (beta-range area: β_Beta-range area_ = − 0.001 ± 0.031, P = 0.97; beta-range peak: β_Beta-range peak_ = − 0.021 ± 0.027, P = 0.44; magnitude of descending coherence: β_Beta-range descending_ = 0.22 ± 3.21, P = 0.95; magnitude of ascending coherence: β_Beta-range ascending_ = 3.68 ± 3.46, P = 0.29). Additionally, no significant associations were found for motor variability (beta-range area: β_Beta-range area_ = 0.055 ± 0.076, P = 0.52; beta-range peak: β_Beta-range peak_ = 0.078 ± 0.065, P = 0.23; magnitude of descending coherence: β_Beta-range descending_ = 7.40 ± 7.77, P = 0.34; magnitude of ascending coherence: β_Beta-range ascending_ = 2.72 ± 8.36 , P = 0.75).

## Discussion

We studied developmental differences in corticomuscular coherence during a visually guided precision grip force-tracing task using source-reconstructed brain activity from EEG data. Magnitudes of beta-band corticomuscular coherence were greater for adults (20–30 yo) compared to children (8–10 yo). This was paralleled by greater levels of descending—but not ascending—directed connectivity for adults compared to children and young adolescents. Effect sizes were small-to-moderate. Our study provides a detailed characterization of developmental differences in corticomuscular functional connectivity during control of low-level force output from the intrinsic hand muscles in a large sample of typically developed children, adolescents and adults.

Comparing corticomuscular coherence in children, adolescents and adults revealed several interesting findings. Characterizing the distribution of peak coherence at the sensor-level for different age groups revealed a similar organization of oscillatory coupling within the corticomuscular system. That is, during the tonic precision grip task, children, adolescents and adults predominantly displayed peak EEG-EMG coherence in the beta range (15–30 Hz). This finding, and earlier evidence of increased beta-range coupling during tonic contractions in humans and monkeys^25^, led us to restrict the DICS source localization procedures to the beta band.

Cortical sources coherent with the EMG activity of the active FDI muscle were found in the precentral and postcentral gyrus of the contralateral sensorimotor cortex (extending into the superior parietal lobule and the middle frontal gyrus). This is in agreement to what has been found in previous studies^41,31^. Peak values of coherence were generally quite low (range 0.001–0.236), but still within the range commonly reported for corticomuscular coherence in both humans (on the sensor-level^12,15^) and non-human primates (using LFPs^13,23,31^). It is a well-known phenomenon that not everyone display magnitudes of corticomuscular coherence that exceed chance levels^51^. The proportion of individuals displaying significant coherence on either their dominant or non-dominant hand amounted to ~ 20% for the entire sample. This percentage is also quite modest, and while it seems lower than what reported when only adults are included^51^, it complies well with what has previously been reported in samples including both children, adolescents and adults^15^. Task-related features such as the gain of the visual feedback^52^ as well as the compliance of the force sensors used during the motor task^53^ can affect coherence levels and this may also contribute to differences between studies in reported incidence.

In the context of developmental differences, we found that adults were characterized by greater levels of coherence compared to children. This was expressed both as differences in the area of beta-range coherence and in the peak values of coherence. Furthermore, these age-related effects were seen when coherence was computed from the average peak location of the sample and when it was computed from the individual peak values. This suggests that it is unlikely that the reported developmental differences were systematically influenced by the fact that cortical time-series were extracted from the grand-averaged peak location in the main analysis.

Coherence in the corticomuscular system is transiently (and oppositely) modulated by skilled motor practice^54,55^ and limb immobilization^56^ suggesting that the corticomuscular system adapts in the face of changes in sensorimotor demands, i.e. with learning or disuse. Here, we demonstrate small-to-moderate developmental differences in functional coupling that could reflect adaptations and tuning of sensorimotor control processes occurring over longer time scales. These results from a large sample of typically developing individuals are well in line with previous studies demonstrating age-related differences in levels of functional cortico-motor coupling in children, adolescents and adults^15,16,18^. Our results are also in agreement with studies showing developmental differences in both spontaneous and movement-related beta power over sensorimotor cortical areas^18,57,58^, suggesting that developmental tuning of beta-range oscillations occurs regionally at the cortical level and in the oscillatory synchronization between cortex and muscle during motor tasks. Coherence may promote efficient communication between distant neuronal populations^11^. The increased levels of corticomuscular coherence could therefore be interpreted as reflecting a greater or more efficient engagement of oscillatory control mechanisms in corticospinal networks during the control of low-level force output in adults compared to children^59,60^.

Corticomuscular coherence has traditionally been considered a motor phenomenon reflecting efferent oscillatory activity descending from the M1 via corticospinal pathways ^24,61,62^, but growing evidence suggests that ascending sensory activity also contributes to the coupling between sensorimotor brain regions and muscle^24,28,29^. Coherent activity between cortex and muscle might thus rather reflect sensorimotor integration processes, constituting a loop in which ascending sensory information interact with descending motor activity and vice versa. One theory is that the descending activity reflect feedforward control processes that predict and probe the state of the system, whereas ascending activity could reflect sensory feedback processes reflecting the actual state of the system^25^. Using measures of directed coherence, we specifically investigated the contribution of descending and ascending processes to coherence in children, adolescents and adults. These analyses revealed that absolute levels of descending (cortex-to-muscle) beta-range coherence were greater in adults compared to children and young adolescents. This was not the case for the ascending part of coherence. Similar developmental shifts in directed corticomuscular connectivity during a force-tracing task involving the ankle muscles have been reported previously^16^. Increases in predictive, feedforward control are generally seen from early to late childhood during both walking and finger movements^3,63^, while decreases in task-related transmission from sensory afferents to motor neurons have been demonstrated to occur at the spinal level concomitantly^64,65^. We suggest that our results support the notion of a shift in the relative reliance on feedforward and feedback control during motor actions between children and adults. However, these interpretations should be weighed in the context of the rather small levels of coherence observed across the entire sample and the small-to-moderate effect sizes reported for the comparisons between age groups.

The implications of these developmental differences in neural control strategies remain unclear. The functional relevance of corticomuscular connectivity to behavior has been extensively debated in the past decades^60,66^. Beta band coherence is prominent during tasks requiring maintained motor outputs^13^, but decreases in magnitude or disappears with actual movement^13,67,68^. This has led to the suggestion that oscillatory activity in the beta-range is involved in maintaining steady motor output^39,69^. We found greater levels of beta-band coherence in adults who were also characterized by performing the task with greater precision and less variability. It therefore seems reasonable to speculate that levels of corticomuscular coherence are associated with the ability to precisely control static force levels. This is in fact also supported by earlier studies showing greater magnitudes of beta-band coherence when precision (or requirements for precision) in a motor task is high (or variability of motor output is low)^20,21,70^. In contrast, no statistical associations were found between measures of coherence and precision grip performance in the present study. This could be related to the substantial variability inherent in measures of coherence between individuals (as seen in the present study, but also specifically investigated in Ushiyama et al.^51^).

Another interesting observation was that levels of corticomuscular coherence were significantly greater on the non-dominant hand compared to the dominant hand, although the effect size was small. Ushiyama et al.^22^ previously reported that corticomuscular coherence was greater for lower-limb muscles compared to upper-limb muscles; and for distal muscles compared to the proximal muscles. Here, we report small between-limb differences in corticomuscular coherence for the same muscle due to limb dominance, with slightly larger levels of coherence reported for the non-dominant compared to the dominant hand. Notably, although these effects were subtle, they were most likely not due to order effects (i.e. that participants in the main experiment always performed the task with the non-dominant hand first) as suggested by a set of control experiments in which the order of the task was reversed. Indeed, no significant effect of order was found in any of the analyses performed. This suggests a task-related modulation of coherence amplitudes relating to the hand used. We speculate that the difference in coherence between hands could be related to the additional demands imposed by using the non-dominant hand to control pinch force. The fact motor variability was higher on the non-dominant hand compared to the dominant hand could indeed indicate that the task was more challenging when it was performed with the less-skilled, non-dominant hand. The corticomuscular system might attempt to overcome this challenge by tuning the coupling efficiency between the cortex and the muscles, which may allow a more efficient binding of sensory and motor information. In line with this idea, greater coherence has previously been observed during challenging dual-task conditions^71^; when requirements for precise control is increased by modulating the gain of the visual feedback^52^; and in visually-guided walking demanding accurate placement of the feet based on visual cues as opposed to normal walking without such requirements^72^. The fact that this task-dependent modulation was observed across different age groups suggests this as a general feature across different stages of typical motor development. However, the effect was only small, and this should be considered when assessing its potential relevance.

Dexterous performance continues to improve from childhood until early adulthood, but the underlying neural mechanisms are not well understood. We demonstrate that oscillatory corticomuscular connectivity is greater in magnitude in adults compared to children younger than 10y. This suggests that functional sensorimotor networks continue to develop through adolescence leading to a strengthening of the functional coupling between the activity of the cortex and of the spinal motor neurons of the hand muscles. We further found that adults were characterized by greater levels of descending connectivity in the corticomuscular system where oscillatory cortical activity leads muscle activity. We propose that this reflect a greater degree of feedforward control of movements with typical development. Collectively, our results help us better understand developmental differences in the neural control of dexterous movements in children, adolescents and adults. This may contribute to our understanding of typical and atypical motor development.

## Supplementary Information

Supplementary Figures.

## Data Availability

Datasets generated and analyzed for this current manuscript are available from the corresponding author upon reasonable request.
