# Supplementary material for: Reorganization of functional and directed corticomuscular connectivity during precision grip from childhood to adulthood
Source: Sci Rep. 2021 Nov 24;11:22870. doi: 10.1038/s41598-021-01903-1 (PMC8613204; doi:10.1038/s41598-021-01903-1)
Supplement: Supplementary file 1 — Supplementary Figures. [file 41598_2021_1903_MOESM1_ESM.docx]

**Reorganization of functional and directed corticomuscular connectivity during precision grip from childhood to adulthood**

Mikkel Malling Beck, Meaghan Elizabeth Spedden, Jesper Lundbye-Jensen

Department of Nutrition, Exercise and Sports, University of Copenhagen, Copenhagen, Denmark

**Supplementary results**

**S1 Associations between log beta area corticomuscular coherence and amount of descending (cortex-to-EMG) and ascending (EMG-to-cortex) components.**

Figure S1 below presents the correlations between the log area and peak of corticomuscular coherence in the beta-range and the decomposed amount of descending (Cortex-EMG; A) and ascending (EMG-Cortex; B) beta-range coherence. Pearson correlation coefficients and corresponding p-values for the correlation analyses are displayed for both the dominant and non-dominant hand.


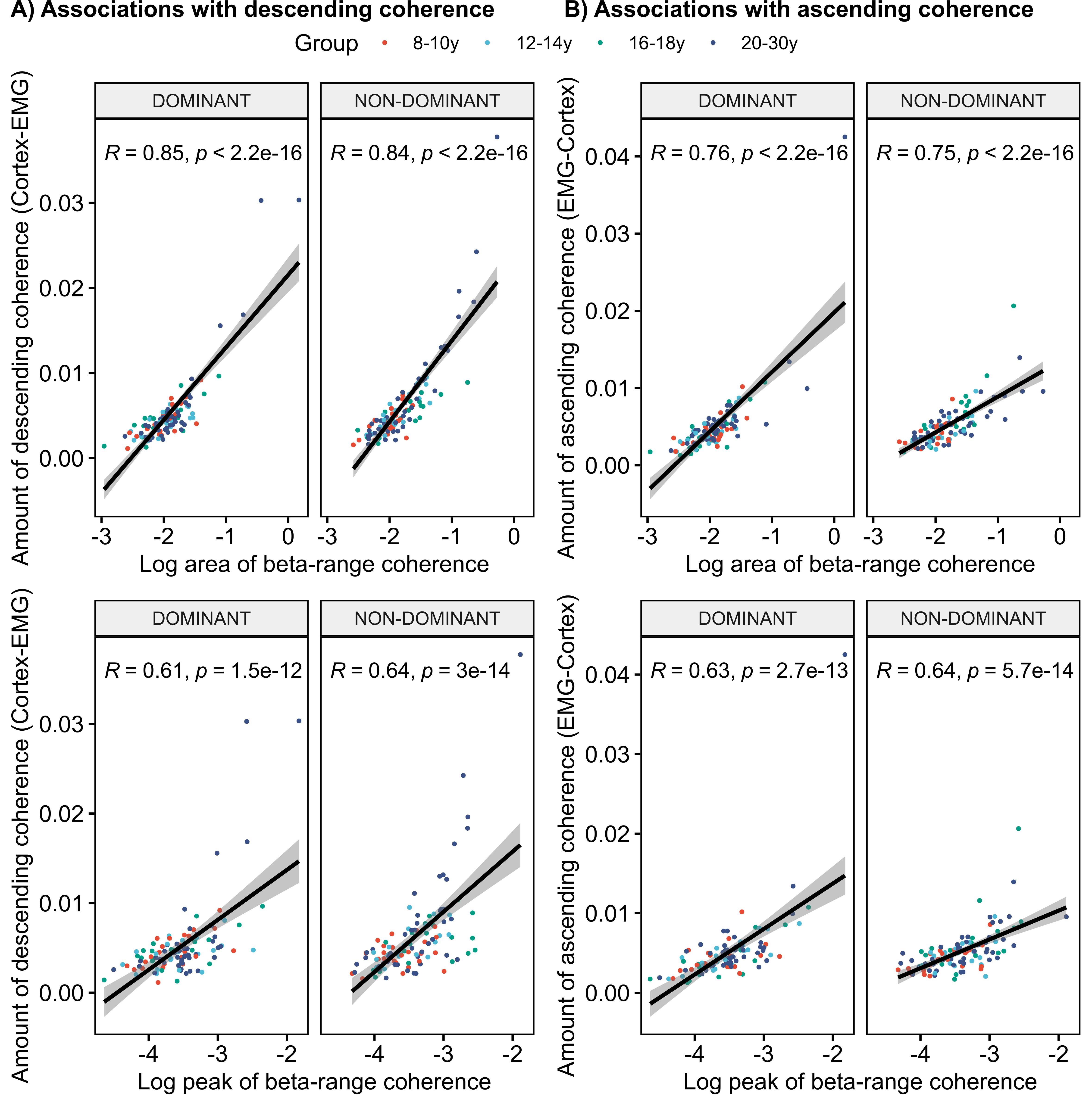


**Figure S1.** Correlations between log area (top) and log peak (bottom) of beta-range corticomuscular coherence and amount of descending (left) and ascending (right) coherence for both the dominant and non-dominant hand.

**S2 Developmental differences and hand difference in corticomuscular coherence at individual peak coherence**

In the main analysis, we used the sample average location of peak corticomuscular coherence to study developmental differences in corticomuscular coherence. These differences in corticomuscular coherence could potentially be confounded by differences in the spatial distribution of sources displaying coherent activity with the active muscle. Therefore, we tested whether age-related differences in magnitudes of coherence were present using the individual peak values from the DICS analysis. This analysis revealed a significant effect of group (F = 6.85, P < 0.001), and subsequent pairwise comparisons suggested that this was driven by 20-30y displaying significantly more corticomuscular coherence than 8-10y (β_20-30y vs 8-10y_  = 0.84 ± 0.20; 95% CI = [0.29 1.39]; P < 0.001; Cohen's d = 0.83) and the 12-14y (β_20-30y vs 12-14y_  = 0.63 ± 0.21; 95% CI = [0.07 1.20]; P = 0.001; Cohen's d = 0.61 and that the 16-18y displayed greater coherence than the 8-10y (β_16-18y vs 8-10y_  = 0.65 ± 0.23; 95% CI = [0.04 1.26]; P = 0.010; Cohen's d = 0.81) (Figure S2). No differences were found in the remaining contrasts (range of p-values 0.09-0.39). There was also a significant effect of hand (F = 11.17; P = 0.001), and this was driven by greater levels of coherence on the non-dominant compared to the dominant hand (β_ND vs DOM_ = 0.28 ± 0.08; 95% CI = [0.11 0.44]; P = 0.001). No significant interaction between group and hand was seen (F = 1.99; P = 0.12).


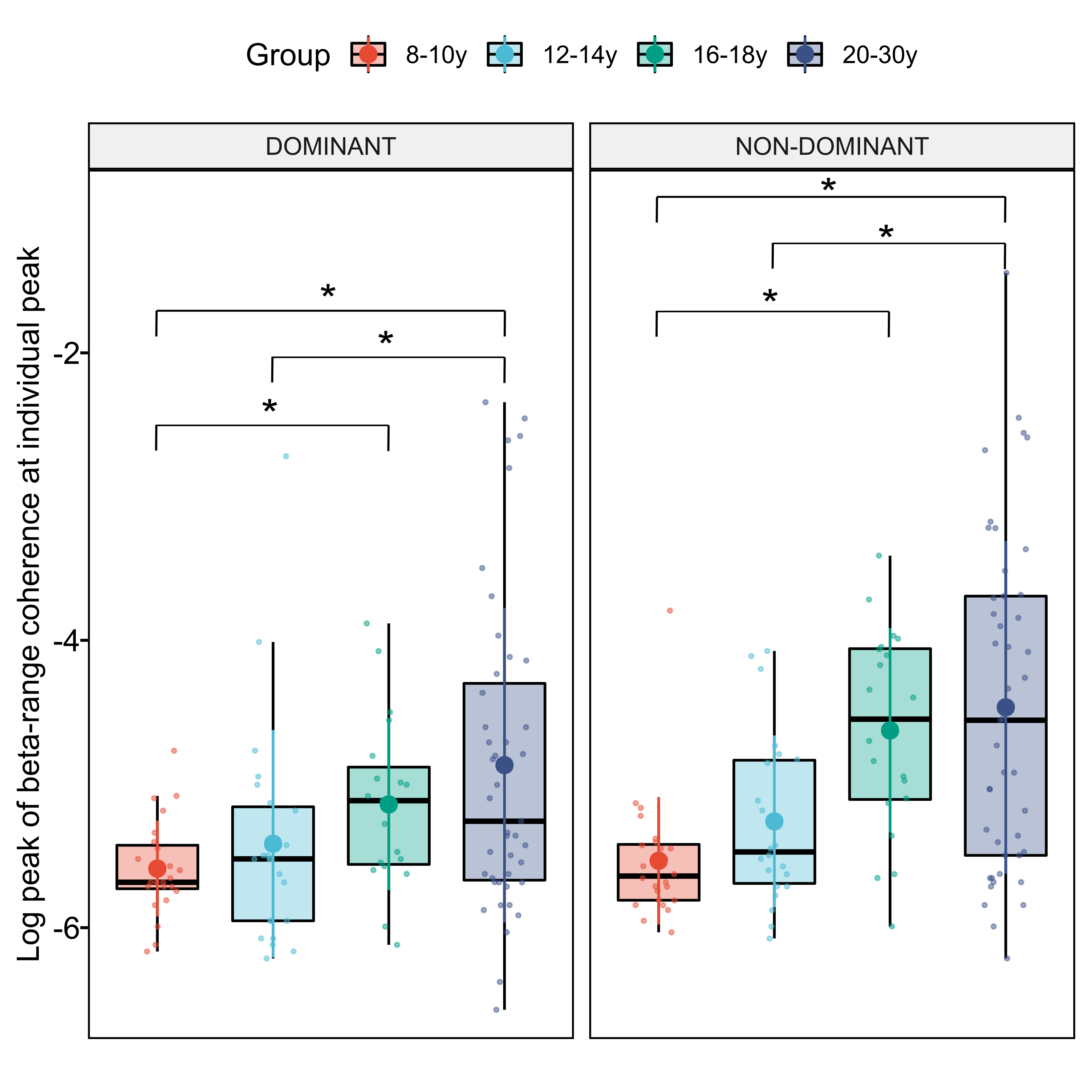


**Figure S2. Peak of corticomuscular coherence on the log-scale at individual peak locations from dynamic imaging of coherent sources (DICS).** Statistical differences between groups across hands are denoted by * (p < 0.05)
